# Patient-Orientated Evaluation of Treatment of Non-Melanoma Skin Cancer with Rhenium-188 Compared to Surgery

**DOI:** 10.3390/healthcare12090921

**Published:** 2024-04-29

**Authors:** Maila I. C. Krönert, Sarah M. Schwarzenböck, Jens Kurth, Martin Heuschkel, Bernd J. Krause, Steffen Emmert, Julia K. Tietze

**Affiliations:** 1 Clinic and Policlinic for Dermatology and Venereology, University Medical Center Rostock, 18057 Rostock, Germany; maila.kroenert@uni-rostock.de (M.I.C.K.); steffen.emmert@med.uni-rostock.de (S.E.); 2 Department of Nuclear Medicine, University Medical Center Rostock, 18057 Rostock, Germany; sarah.schwarzenboeck@med.uni-rostock.de (S.M.S.); jens.kurth@med.uni-rostock.de (J.K.); martin.heuschkel@med.uni-rostock.de (M.H.); bernd.krause@med.uni-rostock.de (B.J.K.)

**Keywords:** rhenium-188, non-melanoma skin cancer, surgery, treatment

## Abstract

Background: Non-melanoma skin cancers (NMSCs) are responsible for up to one-third of all human malignancies. Surgery is usually the treatment of choice, but patients often experience pain during the procedure. Topical rhenium-188 resin skin cancer treatment (RSCT) may be a valid therapeutic alternative. Methods: In this monocentric pilot study, 19 patients suffering from NMSC were treated with RSCT. Most of these patients had also experienced surgery, either because they developed a new NMSC in aftercare, or they had suffered previously from NMSC. Three RSCT-treated patients, who had no exposure to surgery so far, were paired with three matched patients, who had received surgery. We sought to evaluate and compare the patients’ experience with both treatments. A questionnaire assessed patients’ perceptions regarding side effects, aesthetic outcomes, wound care, fear of complications, and personal treatment preferences. Patients evaluated the different parameters of their either RSCT- or surgery-treated lesions on a scale from 0–10. Results: Patients were more afraid of complications before surgery than before RSCT (*p* = 0.04). Treatment with RSCT caused significantly less pain on treatment day (mean 0.56) than surgery (mean 2.32) (0 no pain, 10 maximum pain) (*p* = 0.02) and 14 days after the procedure (mean 0.89 versus mean 2.47) (*p* = 0.02). On day 14, RSCT-treated lesions were also significantly less itchy (mean 0.34) than after surgery (mean 1.50). Most patients were very satisfied with the aesthetic outcome after both RSCT (mean 8.42) and surgery (mean 8.31) (*p* = 0.89). In the case of a new NMSC, the majority of patients who experienced both treatments would rather be treated primarily with RSCT (44%) or would consider both options (31%); only 19% preferred surgery. Conclusion: Patients evaluated RSCT as less painful than surgery. The aesthetic outcomes of both treatments were comparable. For pain-sensitive patients, RSCT might be a preferable treatment option.

## 1. Introduction

Non-melanoma skin cancer (NMSC) is the most frequent of all tumors in the light-skinned population. They are responsible for up to one-third of all human malignancies, and the incidence is still rising [1,2,3]. The World Health Organization stated in 2017 that globally two to three million NMSCs occur every year [4]. NMSCs comprise mostly basal cell carcinomas (BCC) (75%) and cutaneous squamous cell carcinomas (cSCC) (20%) [1,2]. The true disease burden of NMSCs remains unclear and is often underestimated since many cancer registries do not register NMSCs or record only the appearance of the first tumor [2,5,6].

BCC is a slow-growing tumor and very rarely metastasizes [7], but may lead to the destruction of facial sensory organs, causing high morbidity [8]. CSCC may also grow only locally destructive, but it metastasizes much more frequently than BCC. Metastatic cSCC has an annual incidence of approximately 4%, and about 20% of skin cancer deaths are caused by cSCC [9]. The main cause for developing BCC and cSCC is chronic exposure to ultraviolet radiation, which may directly drive the malignant transformation of progenitor cells [10]. Other risk factors include immune suppression, external radiotherapy, or exposure to arsenic [11]. All subtypes of BCC except the superficial subtype as well as invasive cSCC and Bowen’s disease are usually primarily excised, depending on the location using conventional or Mohs micrographic surgery. Mohs micrographic surgery is the gold standard for lesions on the face or hand; more than 95% of the patients are disease-free after 5 years [12,13]. However, pain is a frequent complication of surgery [14], and fear of pain caused by surgery may lead to procrastination of the necessary procedure by the patients [15]. The aesthetic outcome of surgery is usually satisfying, but less so if a skin graft is needed [16]. However, if the tumors are located in sensitive areas such as ears or nose, surgery may also sometimes lead to disfigurement [12,16,17].

Radiotherapy is an efficient alternative treatment for BCC with similar response and recurrence rates to after Mohs surgery but is mainly used for patients not eligible for surgery with more advanced BCC [18]. For patients with more advanced cSCC, external beam radiotherapy and brachytherapy have also shown an increased disease-specific survival and overall survival [11,19].

Application of rhenium-188 skin cancer therapy (RSCT) is a new high-dose brachytherapy for the treatment of NMSC. Rhenium-188 is a high-energy beta-emitting therapeutic radioisotope, which destroys the tumor cell up to 3 mm in depth and leads to activation of the immune system. Patients are usually treated with a one-time topical application of a paste called Re-188-resin. This treatment is not yet part of the clinical routine and is available only in very few centers worldwide. We showed in a recently published study that RSCT is as effective as Mohs surgery. However, the follow-up time was only 12 months, and more data need to be acquired over the next years [20]. The most common side effects of the treatment are radiodermatitis after 14 days (97.5% of the lesions) and hypopigmentation after 12 months (49% of the lesions); only 12.5% of the lesions were reported to be painful at any time point [20].

Since RSCT is a valid alternative to surgery, we were interested in how the patients perceived both treatments in comparison. To explore this question, we conducted a follow-up study with the RSCT-treated study patients.

## 2. Materials and Methods

### 2.1. Study Design and Ethics Approval

All procedures performed in this study were in accordance with the 1964 Declaration of Helsinki and its subsequent amendments. This study was approved by the local ethics committee; a first ethics vote approved the primary study, in which we questioned the patients treated with RSCT (A2020-178, date 30 July 2020), and a second ethics vote approved questioning the patients about their surgery (2023-0061, date 23 April 2023). Patients were eligible if they had histologically confirmed BCC or cSCC, were at least 18 years old, and had been treated with RSCT for basal cell or squamous cell carcinoma (Rhenium-SCT^®^/OncoBeta^®^ study) [20] and/or received surgery as a treatment for NMSC in the last 4 years. Treatment with RSCT took place in the department of nuclear medicine. Patients were treated only once with RSCT [20]. RSCT was developed by Oncobeta^®^ GmbH, Munich, Germany. The study was set up as an add-on to the preliminary pilot study [20] for the currently recruiting registered EPIC study NCT05135052.

### 2.2. Setting and Participants

Between November 2020 and June 2023, we interviewed 22 patients. Nineteen patients received RSCT in the context of the rhenium-188 resin treatment study [20]. Sixteen of the nineteen RSCT-treated patients had received surgery before or after the treatment with RSCT to treat NMSC in comparable locations to the RSCT-treated locations. Three of these patients had not experienced surgery so far and were matched for age and location with three other patients who had been treated only with surgery, and their evaluations of the lesions were paired. The tumor size ranged from 0.04 cm^2^ up to 16.8 cm^2^ (median 1.25 cm^2^) [20]. The treated area was the face including the front, nose, and cheek; head including the temple, subauricular region, parietal region, and ear; trunk including breast, décolleté, back, and abdomen; and lower extremities including thigh, crus, and foot. About 33% of the NMSC had been unsuccessfully treated previously with imiquimod, with surgery, or diclofenac gel and curettage [20]. In the previous study, follow-up visits were scheduled at 14 days, 4 months, and 12 months after RSCT; the patients were therefore asked for an evaluation of their side effects retrospectively at these time points. The survey was conducted with a self-created “Non-melanoma skin cancer therapy questionnaire” (Supplementary Materials).

### 2.3. Therapy Details

A Re-188 resin-filled carpoule, a measurement station (dose calibrator), and an application system with a brush for manual application were used for Rhenium-SCT^®^, Skin Cancer Therapy, Oncobeta^®^ GmbH, Germany. Rhenium has a half-life of 17 h; therefore, first the radioactivity of Re-188 resin was measured and the duration of the application was calculated, taking into account the activity, the thickness of the tumor, and the size of the lesion, assuming a standardized empiric mean absorbed target dose of 50 Gy to the deepest point of the tumor [20,21,22,23]. The lesions were covered with a 7 μm foil to prevent direct contact of the substance with the skin and to facilitate removal of the substance after the termination of treatment. Afterward, the substance was applied. After the treatment the remaining radioactivity in the carpoule was measured and the foil was removed and properly disposed of. Radiation protection of patients and staff was ensured the entire time. Handling and release of radioactive waste from regulatory control were performed according to the requirements of local authorities [20].

The surgical procedures were performed by experienced dermatologic surgeons in the hospital operating rooms under sterile conditions. For all surgical procedures, informed consent was obtained before surgery.

### 2.4. Details of the Questionnaire

The questionnaire consisted of 22 questions and was written in German. The questions were divided into general and treatment-specific questions. General questions concerned age, gender, relationship status, employment status, physical activity in leisure time, time after the first diagnosis of the NMSC, and previous and current therapies of the patient. In the part with the specific questions, the patients were asked to grade their side effects, such as pain, itching, or burning to different time points as well as the inconvenience with wound care on a scale from 0–10 (0 no not at all, 10 very much). Patients were also asked to evaluate the aesthetic outcome on a scale from 0–10 (0 very bad, 10 perfect), their fear of the procedure, and their fear of complication on a scale from 0–10 (0 not at all, 10 very much).

Only patients who had experienced both treatments were questioned about which treatment they would prefer if they developed a new NMSC.

### 2.5. Validation of the Questionnaire

After creating the questionnaire, it was validated by ten independent test subjects. The test subjects were asked to evaluate the questionnaire regarding simplicity, comprehensibility, and relevance on a scale from 0–5 (0 bad, 5 very good). The grading for simplicity was (5.0 ± 0), for comprehensibility (4.7 ± 0.3), and for relevance (4.8 ± 0.2), which was considered to be acceptable.

### 2.6. Data Analysis

Statistical analysis was performed using GraphPad Prism software (version 5.01, GraphPad Software Inc., San Diego, CA, USA). Analysis of differences between the patients was performed using a paired *t*-test. A *p*-value of <0.05 was considered significant (* *p* < 0.05, ** *p* < 0.01, *** *p* < 0.001).

## 3. Results

### 3.1. Population Characteristics

Between November 2020 and April 2023, we included 22 patients in the study. The median age was 83 (39–90) years, and 60% were females. A total of 68% of the patients lived in a stable relationship, and 79% were in retirement. Most patients in our study suffered from BCC (65.8%), followed by Bowen’s disease (18.4%) and invasive cSCC (15.8%). Nineteen of the patients were treated with RSCT, and three patients only with surgery. Half of the patients needed surgery in the months after the end of the study to treat a newly diagnosed NMSC, and half of these patients had an operation on an NMSC in the months before the possibility to participate in the study occurred. The median time between surgery and RSCT was 5.3 months. The lesions were located on the head including the ear and face (63%), on the trunk (26%), and on the lower extremity (11%) (Table 1). The treatment took place between July 2019 and March 2022.

The treatment modalities were independent of the kind of the tumor since all NMSCs were treated with RSCT in the study, and outside of the study all NMSCs on the face and head were excised in Moh’s surgery and all NMSCs on the lower leg or trunk were removed in a single excision. Most patients experienced a second NMSC in a similar area to the first lesion (Table 2).

### 3.2. Treatment Decision

We were curious why the patients chose to participate in a study with a new compound instead of just receiving surgery. The main reason for participating in the study was the wish to avoid pain and complications of the surgery (84%). In most of these cases (47% of all patients), this decision was strongly supported by the dermatologist since the lesions were either large and located on sensitive areas such as the nose, tibia, or ear or the patient had received multiple prior operations, which would have led to very complicated wound closure. Other reasons for participation in the study were the desire for close supervision, which was provided for patients participating in the study (11%), or interest in a new treatment option in case of a valid medical indication for both treatments (5%).

### 3.3. Fear of the Procedure

**Figure 1:**
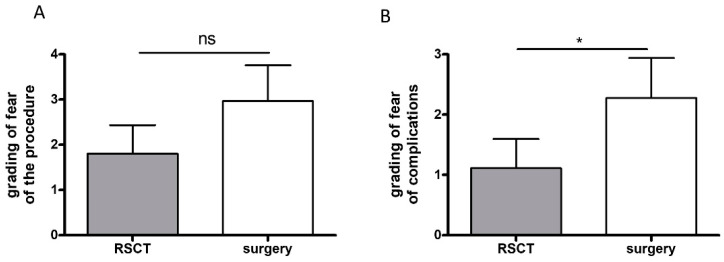
Assessment of fear of the procedure itself and fear of complications: Patients treated with RSCT feared the procedure less (mean 1.81) than patients receiving surgery (mean 2.97), but the difference was not significant (*p* = 0.16) (**A**). Patients were significantly more concerned about complications before surgery (mean 2.28) than before RSCT (mean 1.11) (*p* = 0.04) (* *p* < 0.05) (**B**).

Next, we assessed the patients’ fear of the different procedures, since fear of the procedure itself or of later occurring complications may lead to procrastination of the treatment. The study patients were asked to grade their general fear of the specific therapy itself and complications (0, no fear; 10, maximum fear). The patients reported that they were less afraid (mean 1.81) of RSCT than of surgery (mean 2.97), but the difference was not significant (*p* = 0.16) (Figure 1A). However, being asked more specifically about fear of complications, the patients stated that they were significantly more concerned about complications before surgery than before treatment with RSCT; 44.4% experienced fear of complications before surgery, but only 27.8% were afraid of complications before RSCT (mean 2.28 versus 1.11), (*p* = 0.04, Figure 1B). Over twelve months after the procedure, the patients were asked whether they evaluated their prior concerns as justified (0 not justified, 10 completely justified); 27% of patients felt their fears came true after surgery (mean 1.39), but only 17% of the patients reported the same after RSCT (mean 0.69), but the difference was not significant (*p* = 0.09).

### 3.4. Reported Adverse Events

**Figure 2:**
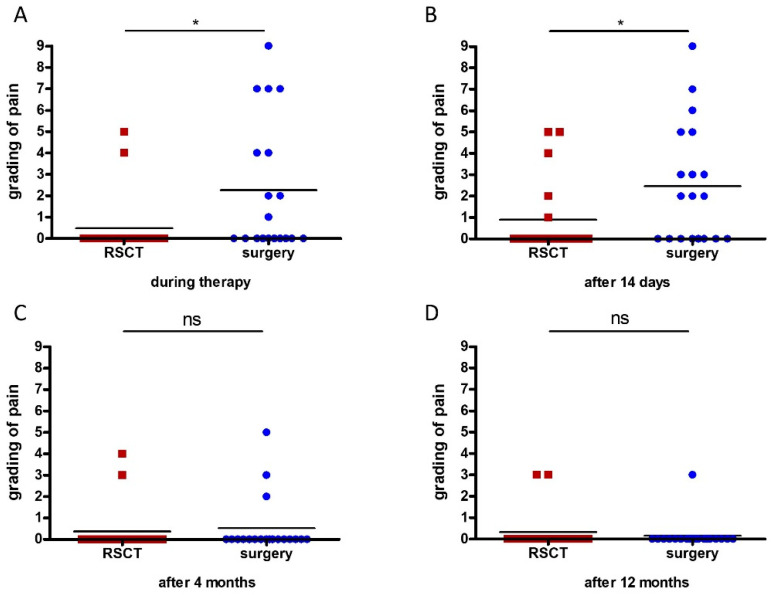
Grading of pain at different time points after the treatments: Patients reported more pain during surgery (mean 2.32) compared to treatment with RSCT (mean 0.56) (*p* = 0.02) (**A**). Fourteen days after the procedure, the operated lesions were more painful (mean 2.47) than the RSCT-treated lesions (mean 0.89) (*p* = 0.02) (**B**). The reported pain levels four months (**C**) after treatment with RSCT (mean 0.37) or surgery (mean 0.53) (*p* = 0.18) and 12 months (**D**) after treatment with RSCT (mean 0.37) or surgery (mean 0.16) (*p* = 0.33) did not differ between both treatments (* *p* < 0.05).

**Figure 3:**
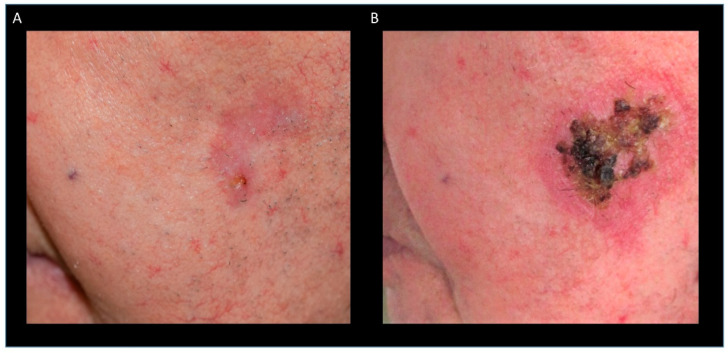
Radiodermatitis after RSCT: patient with a BCC on the cheek before RSCT (**A**) and 14 days after RSCT with radiodermatitis grade II (**B**).

Pain is a major reason for patients to avoid procedures. We therefore asked the patients to grade their pain level during and after their surgery and their RSCT (0, no pain; 10, maximum pain). Since all patients except the three randomized pairs experienced both RSCT and surgery, we compared most patients to themselves. The patients reported to have experienced very little pain in general; however, the patients experienced still significantly more pain during surgery (mean 2.32) compared to RSCT (mean 0.56) (*p* = 0.02) (Figure 2A). Since the application of the Rhenium-resin and its action is painless, we expected that result. However, even 14 days after surgery, the operated lesions were still significantly more painful (mean 2.47) than the lesions treated with RSCT (mean 0.89) (*p* = 0.02) (Figure 2B), which was very surprising since after 14 days almost all lesions (97.5%) treated with RSCT show clinically distinct signs of radiodermatitis [20] (Figure 3). But even though the lesions looked painful, the patients felt little pain. The reported pain levels for the lesions four months after RSCT (mean 0.37) or surgery (mean 0.53) (*p* = 0.18) and 12 months after RSCT (mean 0.37) or surgery (mean 0.16) (*p* = 0.33) did not differ (Figure 2C,D).

**Figure 4:**
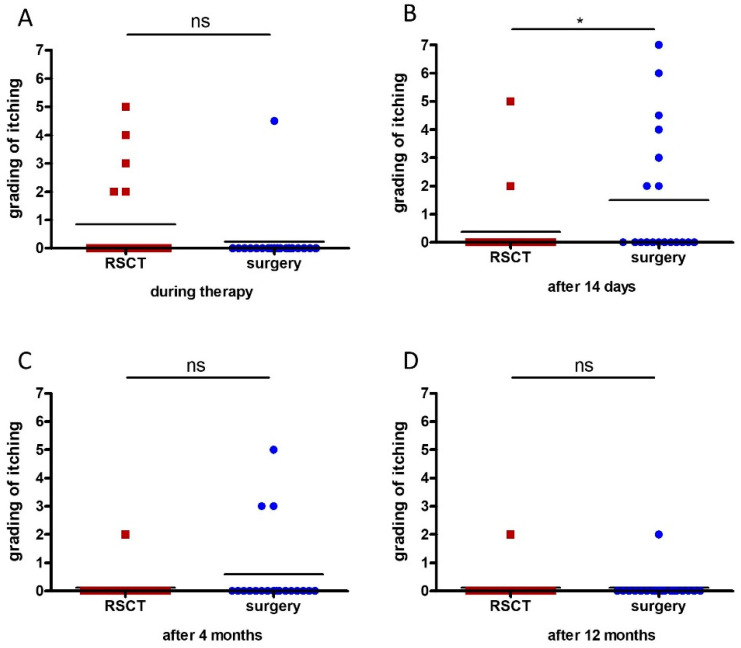
Grading of itching at different time points after the treatment. During RSCT, more patients experienced itching than during surgery, but the difference was not significant (0.22) (**A**). After 14 days, the operated lesion itched significantly more than the RSCT-treated lesions (*p* = 0.02) (**B**). Four months after therapy, the lesions treated with RSCT were slightly less itchy than after surgery, but the difference was not significant (*p* = 0.20) (**C**). After 12 months, very little itching was experienced on all the lesions (mean 0.11) (*p* = 1.00) (**D**) (* *p* < 0.05).

Itching may also increase the discomfort of patients after treatment; therefore, we assessed itching of the lesions after the two different treatments using a scale from 0 to 10 (0 no itching, 10 maximum itching). Patients reported slightly more frequent itching during RSCT treatment (0.84) than during surgery (0.24), but the difference was not significant (0.22) (Figure 4A). Fourteen days after treatment, however, the operated lesions were significantly more itchy (mean 1.50) than the RSCT-treated lesions (mean 0.37) (*p* = 0.02) (Figure 4B). Four months after therapy, the lesions treated with RSCT (mean 0.11) were slightly but not significantly less itchy than after surgery (0.58), (*p* = 0.20) (Figure 4C). After 12 months, basically no itching was experienced anymore either after RSCT (mean 0.11) or surgery (mean 0.11) (*p* = 1.00) (Figure 4D).

We also asked the patients to evaluate a burning sensation as a specific quality of pain under or after treatment with RSCT or surgery. The mean level of a reported burning sensation was very low at any time point (mean < 0.80) and at no time point differed significantly between the lesions treated with surgery or RSCT.

### 3.5. Assessment of Restriction after Therapy

After RSCT, most patients develop radiodermatitis with erythema, scabs, and erosions, which requires specific wound care [20].

**Figure 5:**
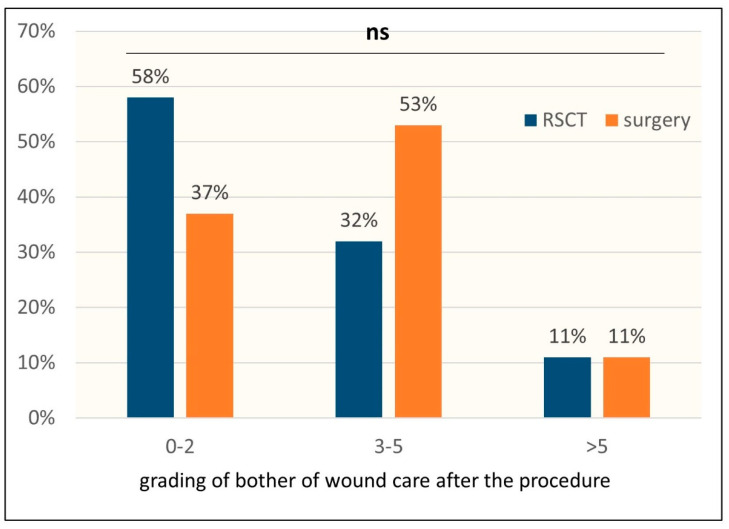
Grading of the bother with wound care: Patients graded their trouble with wound care after therapy on a scale from 0 (not all) to 10 (very much). Most patients were not or only slightly bothered (0–2) by caring for the wound after RSCT, and most patients after surgery were moderately bothered (3–5) with wound care. The overall difference in the grading of the bother of wound care, however, was not significant (*p* = 0.48). The patients reported only marginal restriction in their usual activities after RSCT (mean 1.39) as well as after surgery (1.53) (*p* = 0.83).

After surgery, bathing and swimming are not recommended; furthermore, the wound needs disinfection and bandages and the suture has to be removed after a defined amount of time. In case of complications such as infections or wound dehiscence, the wound will need even more extensive care. Caring for the wound after therapy may hamper the usual activities in working or leisure time after both treatments. Since therefore both treatments may lead to wounds in need of care, we assessed the grade of how much the patients were bothered by taking care of the RSCT or surgery-inflicted wounds (0 not at all, 10 very much). The RSCT-treated lesions did not or only slightly bothered most of the patients (58%), whereas after surgery the majority of the lesions required more wound care, and the patients were moderately affected by it (53%). Eleven percent of the lesions after RSCT and after surgery required extensive care. The overall difference in the grading of the bother of wound caring was not significant between the patients treated with RSCT (mean 2.37) or surgery (mean 2.72) (*p* = 0.48) (Figure 5).

### 3.6. Evaluation of Aesthetic Outcome

**Figure 6:**
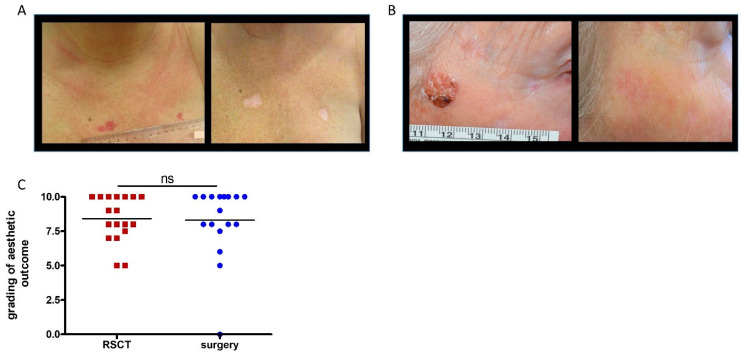
Evaluation of aesthetic outcome of the treatments: RSCT may cause hypo- or hyperpigmentation (**A**) or, in the best case, no scarring or changes in the pigmentation of the skin (**B**). There was no significant difference in the overall aesthetic evaluation of the lesions after RSCT or surgery (*p* = 0.89) (**C**).

Surgery may cause only an almost invisible scar or, in the worst case, extensive scarring or even disfigurement. RSCT may also cause slight scarring, and in about 49% hypo- or hyperpigmentation occurs, but in the best case, invisible or only barely visible changes in the skin are observable (Figure 6A,B) [20]. We were therefore interested in how the patients evaluated the aesthetic outcome of their differently treated lesions (0 not satisfied at all, 10 very satisfied). The majority of the lesions (72%) that were treated with RSCT and also the majority of lesions after surgery were graded to have a very satisfying aesthetic result (8–10). Seventeen percent of the lesions treated with RSCT and eleven percent of the lesions treated with surgery were evaluated as aesthetically good (6–7). Eleven percent of the RSCT-treated lesions and 6% of the operated lesions were rated as acceptable (5). None of the lesions that have been treated with RSCT and one (6%) of the lesions treated with surgery were graded very badly (0). The overall difference between RSCT (mean 8.42) and surgery (mean 8.31) was not significant (*p* = 0.89) (Figure 6C). Analysis of the level of satisfaction concerning the location of the lesions revealed that the RSCT-treated lesions, as well as the lesions after surgery, were graded better if located on the face including the ear (RSCT mean 9.33, surgery mean 9.19) and body (RSCT mean 8.75, surgery 9.33) than with the result on the head excluding the face (RSCT mean 7.50, surgery mean 5.33) and the lower extremity (RSCT mean 7.67, surgery mean 5.00), but the difference of the grading between the localization of the lesions was not significant for RSCT (*p* = 0.29) or for surgery (*p* = 0.08). There was also no significant difference in the evaluation of the aesthetics of the four different locations between RSCT and surgery (*p* = 0.63).

### 3.7. Treatment of Choice

Only the patients who experienced both treatments (*n = 16*) themselves were asked whether they would prefer surgery or RSCT if they developed a new NMSC.

**Figure 7:**
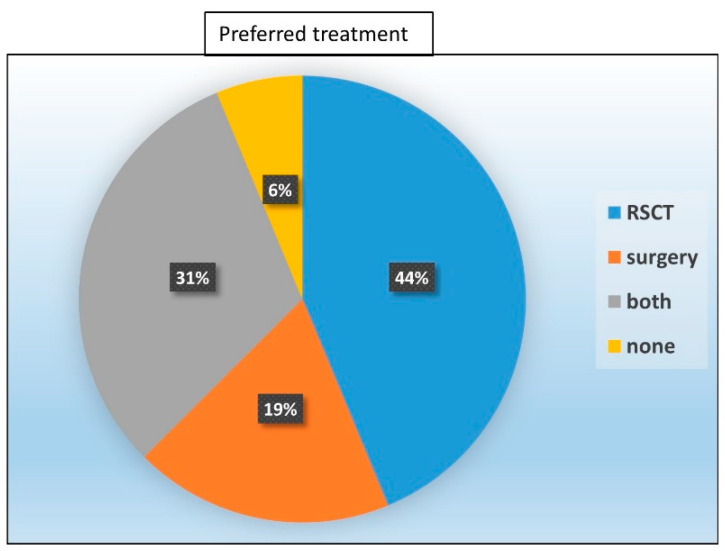
Treatment of choice in case of a new NMSC: Most patients chose RSCT (44%) as treatment in case of a new NMSC. Almost one-third of the patients (31%) would consider both options, 19% would rather have surgery, and one patient (6%) would rather not be treated at all.

Most of these patients chose RSCT (44%) as the favorable future treatment option. Almost one-third of the patients (31%) would consider both options depending on the localization of the lesion and the recommendation of the treating physician, 19% would rather have surgery than RSCT again, and one patient (6%) would decline any treatment at all (Figure 7).

## 4. Discussion

Up-to-date surgery, especially Mohs surgery, is the standard treatment of invasive NMSCs. The clearance rate of >95% is very convincing [20]. Although radiation therapy has shown control rates of 75–100% in early-stage BCC and SCC, the role of radiotherapy has significantly decreased in the treatment of NMSCs with the emergence of Mohs surgery so far [24]. Currently, it is mainly recommended as a primary treatment method if surgery is contraindicated or as adjuvant treatment if clear margins are not accomplishable [24]. RSCT is a new radiotherapeutic treatment option for invasive NMSCs up to 3 mm in thickness and offers an alternative to surgery, especially since the response rate of 95–98% complete responses after a single treatment is comparable to the results after Mohs surgery, as we and others have shown [20,22]. So far, the treatment is only available in very few centers or studies worldwide, and very little data on this treatment have been published. Here, we asked patients who had experienced RSCT and surgery to evaluate their experience with both treatments. To our knowledge, this is the first study of patient-reported outcomes comparing RSCT and surgery.

Even though surgery is often the medically best choice to treat NMSC, in specific cases, an alternative treatment option may be useful, such as if the tumor is localized on sensitive areas such as the ear or nose, where reconstructive surgery may be challenging or the patient has already relapsed after surgery or if the patient is simply afraid of the procedure. Patients frequently delay the visit to the treating physician to receive a final diagnosis and treatment after the first clinical occurrence of NMSC; they avoid the treatment most likely out of fear: Alam et al. showed that the majority of patients (67.6%) waited 6 months on average until they presented to a physician after they first noticed the NMSC themselves, and of these patients, 9.5% waited even 3 years or longer [15]. Patients who waited longer were significantly more concerned about disfigurement or scarring after surgery [15], but waiting also leads to tumor growth, increasing the risk of these complications. We also confirmed the fear of surgery in our study patients: The majority (84%) wanted to participate in the study to avoid pain and complications of the surgery, they reported being more afraid of complications receiving surgery than treatment with RSCT (*p* = 0.04, Figure 1).

The fear of pain is justified since pain is a frequent complication under and after Mohs surgery. In a recent study, between 37% and 44% of the patients experienced moderate pain and 9.5–12.5% even severe pain associated with local anesthetic injections [14]. Since no local anesthesia is needed with RSCT, we expected less pain on the treatment day, and non-surprisingly patients reported significantly less pain under RSCT (mean 0.56) than under surgery (mean 2.32) (*p* = 0.02). A survey of pain after Mohs surgery conveyed that 52% of the patients needed pain medication on the day of surgery; the mean pain level was 2.34 on the WongBaker FACES scale, which is a 0-to-10 scale using descriptive faces to assess pain [25], which is consistent with the pain level in our study.

In almost all patients, RSCT leads to acute radiodermatitis after 14 days, which may also cause pain. It has been reported that the pain level after surgery quickly declines after 4 days [25]. We, therefore, anticipated more pain after 14 days in the RSCT-treated lesions compared to the surgically removed lesions. Our patients, however, experienced after surgery stable pain over the days with a mean level of 2.47 on day 14 and reported on the other hand very little pain on the RSCT-treated lesions (mean 0.89) despite radiodermatitis (*p* = 0.02), which was unexpected. It has been described that patients with prior skin cancer removal have increased postoperative anxiety, which is associated with worse postoperative pain [26]. Almost half of our patients had experienced prior skin cancer removal, which may contribute to the higher grading of the pain level over the first 14 days after surgery.

As mentioned above, disfigurement is a frequent fear before surgery, since surgery may lead to loss of tissue and scarring. The most frequent long-term side effect of RSCT is hypopigmentation (49%), which may be ostentatious specifically in darker skin types [20]. Interestingly, in the end, there was no difference in the patient’s aesthetic evaluation of the outcome of both lesions. The patient’s evaluation of the aesthetic outcome of the operated lesions was mostly, with one exception, very good, which is consistent with published data, in which the satisfaction rate of the patients after Moh’s surgery is usually high [16,17]. Treatment with RSCT led to aesthetically similar results, and patients were also very content with the outcome; there was no significant difference in the aesthetic outcome of RSCT- (mean 8.41) and surgery- (8.31) treated lesions in the overall evaluation of the patients.

Both treatments proved to be effective and yielded comparable aesthetic evaluations; the main difference in our study was the lower pain levels after RSCT compared to surgery. However, this seemed to be very relevant for the perception of the treatment since 44% of the patients who experienced both RSCT and surgery chose RSCT as the preferable first-line treatment in the case of a new NMSC and only 19% of the patients preferred surgery.

The patients in our cohort who would choose RSCT as the first line treatment if a new NMSC occurred were in a median of 85 years of age. The incidence of NMSCs increases with age and is commonly found in nursing homes and geriatric units [27]. Mohs micrographic surgery entails multiple time-consuming surgical and histological examinations for each patient and may lead to longer hospitalization. It has therefore been questioned in the literature whether to treat asymptomatic NMSC at all since it will probably not affect a patient’s life expectancy. However, since untreated NMSC may cause local destruction and disfigurement and old patients may live to be even older, it has been agreed that surgical excision of NMSC in older adult patients is indicated in most situations [27]. Older patients with NMSC, in particular, may benefit from a tailored treatment plan based on current available data for NMSC [28]. Therefore, specifically for older patients, RSCT may be in some cases the more suitable treatment, since no hospitalization is needed, it is painless in most cases, and the duration of the treatment is relatively short. Often it takes only about an hour [20], and older patients seem to prefer this treatment.

A limiting factor of the analysis is the fact that both lesions the patients compared on themselves were not identical either in size or location, but most of the time in similar anatomic regions. We compared lesions on the trunk with lesions on the lower leg, since the surgical approach was the same; however, this decision may be debatable. Furthermore, a time gap existed between both treatments, which may lead to different evaluations; half of the patients who experienced both treatments received surgery ahead of and half after RSCT.

## 5. Conclusions

RSCT as a treatment for NMSC causes significantly less pain than surgery. The aesthetic outcomes of both treatments are comparable, as evaluated by the patients. Patients feared RSCT treatment significantly less than surgery. The availability of RSCT as an alternative treatment option may therefore decrease the time until some patients seek treatment, which would be beneficial.

## Figures and Tables

**Table 1:** Demographics and tumor data.

patient data	number of patients	22
	sex	f: 60% m: 40%
	skin type	Caucasian
	age	39–90 median: 83
specific tumor data	number of lesions	38
	size cm^2^	0.09–9.28 median: 1.4
	Kind of NMSC	
	Bowen’s disease	18.4%
	BCC	65.8%
	cSCC	15.8%
	localization	
	face	29%
	ear	5%
	head without face	29%
	trunk	26%
	lower extremity	11%

Data of the demographics of the 22 participants and the number, size, subtype, and distribution of the treated NMSCs.

**Table 2:** Localization of the compared lesions in the same and/or the randomized patients; grey shades mark the paired patients.

Age	Gender	Kind of Tumor Treated with RSCT	Localization Lesion RSCT	Kind of Tumor Treated with Surgery	Localization Lesions Surgery
88	female	SCC	face	BCC	face
74	female	BCC	trunk	Bowens disease	face
80	female	Bowen’s disease	lower leg	BCC	trunk
87	female	BCC	lower leg	SCC	trunk
85	female	SCC	face	BCC	face
54	male	BCC	head		
55	male			BCC	head
90	male	Bowen’s disease	head	BCC	face
89	female	BCC	face	BCC	trunk
88	male	Bowen’s disease	face	SCC	face
55	female	BCC	trunk	BCC	trunk
86	male	BCC	trunk	BCC	trunk
60	female	BCC	face		
59	female			BCC	face
87	female	Bowen’s disease	head	BCC	head
89	male	Bowen’s disease	head		
80	male			BCC	head
74	male	BCC	trunk	BCC	trunk
80	male	SCC	ear	SCC	ear
82	male	BCC	lower leg	Bowen’s disease	lower leg
89	female	BCC	head	BCC	head
84	male	BCC	head	BCC	head

## Data Availability

The datasets generated during and/or analyzed during the current study are available from the corresponding author upon reasonable request.

## Supplementary Materials

The following supporting information can be downloaded at: https://www.mdpi.com/article/10.3390/healthcare12090921/s1, Questionnaire for the therapy of non-melanoma skin cancer.

## Abbreviations

BCC: basal cell carcinoma, NMSC: non-melanoma skin cancer, cSCC: cutaneous squamous cell carcinoma, RSCT: rhenium-188 resin skin cancer treatment.
