# Supplementary material for: Patient-Orientated Evaluation of Treatment of Non-Melanoma Skin Cancer with Rhenium-188 Compared to Surgery
Source: Healthcare (Basel). 2024 Apr 29;12(9):921. doi: 10.3390/healthcare12090921 (PMC11083370; doi:10.3390/healthcare12090921)
Supplement: Supplementary file 1 [file healthcare-12-00921-s001.zip › healthcare-2927545-supplementary.pdf]

Supplementary Materials

Questionnaire for the therapy of non-melanoma skin cancer

First of all, thank you very much for your willingness to participate in this survey. The following questionnaire asks questions about your therapy against white skin cancer.

Answering this questionnaire is voluntary and will only take a few minutes. Please read the questions carefully and tick the answers that apply to you. The questionnaire comprises a total of **5 pages**. Your information will of course be treated **confidentially** and will only be processed **in anonymous** form. If you need help filling out the questions, please contact Ms. Krönert by e-mail: maila.kroenert@uni-rostock.de, telephone number: 015234102530.

*First, we ask you to answer a **few general questions**.*
(Please tick the appropriate box and, if necessary, add your own handwritten answers in the free text)

Age: ________________years

Gender: ☐ female ☐ male

Relationship status:
☐ single
☐ in a steady partnership

How would you describe your current employment status?
☐ Part-time job (up to 34 hours/week)
☐ Full-time job (35-42 hours/week)
☐ Full-time job (more than 42 hours/week)
☐ retired / not able to work
☐ no occupation, looking for work
☐ No occupation, not looking for work (e.g. parental leave, student)

Do you do sports in your free time?
☐ no
☐ yes, less than 1 hour/week
☐ yes, more than 1 hour/week

How long has your white skin cancer been known?

Selection: ☐ 1 year ☐ 2 years ☐ > 2 years

What prior therapies have you received regarding your white skin cancer?

Selection: ☐ Laser therapy ☐ Cryotherapy ☐ Creams ☐ others ☐ none

Which of the therapies listed below have you received?
Please tick all the applicable boxes.

Selection: ☐ OP ☐ Rhenium-SCT®

Now follow ***specific questions*** *about your final therapy against white skin cancer.*
Tick the number you feel is correct on the corresponding scales from 0-10.

Pain:

How severe was the **pain under therapy/treatment?**

0 none — 10 Very strong

How severe was the **pain 14 days after therapy?**

0 none — 10 Very strong

How severe was the **pain 4 months after therapy?**

0 none — 10 Very strong

How severe was the **pain 12 months after therapy?**

0 none — 10 Very strong

Itching:

How much **itching** did you experience **during therapy/treatment?**

0 Not at all — 10 Very much

How much **itching** had you felt **14 days after therapy?**

0 Not at all — 10 Very much

How much **itching** had you felt **4 months after therapy?**

0 Not at all — 10 Very much

How much **itching** have you experienced **12 months after therapy?**

0 10

Not at all Very much

Burning:

Did you experience **burning of the skin during therapy?**

0 10

Not at all Very much

Did you feel **burning in the skin 14 days after** the **therapy?**

0 10

Not at all Very much

Did you feel any **burning of the skin 4 months after** the **therapy?**

0 10

Not at all Very much

Did you feel any **burning of the skin 12 months after** the **therapy?**

0 10

Not at all Very much

Have you experienced any other side effects while on treatment, if so what are they?____

Wound care:

How much were you bothered by the **care of your wound after treatment?**

0 10

Not at all Very much

Aesthetics/Cosmetics:

How satisfied are you with the final cosmetic result of your skin lesion after therapy?

0 — 10

Not satisfied at all — Very satisfied

If a new white skin cancer lesion appeared, would you perform the treatment again?

0 — 10

No definitely — Yes absolutely

Quality of life:

How much did you **feel limited by therapy in your free time?**

0 — 10

Not at all — Very much

How much have you **felt affected by therapy in the performance of your job/job-like tasks?**

0 — 10

Not at all — Very much

Mental stress:

How **afraid** were you of the **therapy application?**

0 — 10

Not at all — Very much

How **afraid** were you **of complications?**

0 — 10

Not at all — Very much

How justified do you think the **fear of therapy** is **now?**

0 — 10

Not justified at all — Very justified

How justified do you think the **fear of complications** is **now?**

0 — 10

Not justified at all — Very justified

If you had to decide again to treat your white skin cancer, which treatment option would you choose?

Selection:

☐ Rhenium-SCT®	☐ OP	☐ neither of the two therapies	☐ both therapies

You have now reached the end of the questionnaire. Thank you for your participation.
